# Cerebellar and Cortical Correlates of Internal and External Speech Error Monitoring

**DOI:** 10.1093/texcom/tgab038

**Published:** 2021-05-31

**Authors:** Elin Runnqvist, Valérie Chanoine, Kristof Strijkers, Chotiga Pattamadilok, Mireille Bonnard, Bruno Nazarian, Julien Sein, Jean-Luc Anton, Lydia Dorokhova, Pascal Belin, F- Xavier Alario

**Affiliations:** Aix-Marseille Université, CNRS, LPL, Aix-en-Provence 13100, France; Aix-Marseille Université, CNRS, LPL, Aix-en-Provence 13100, France; Institute of Language, Communication and the Brain, Aix-en-Provence 13100, France; Aix-Marseille Université, CNRS, LPL, Aix-en-Provence 13100, France; Aix-Marseille Université, CNRS, LPL, Aix-en-Provence 13100, France; Aix-Marseille Université, Inserm, INS 13005, Marseille, France; Centre IRM, Marseille 13005, France; Aix-Marseille Université, CNRS, INT 13005, Marseille, France; Centre IRM, Marseille 13005, France; Aix-Marseille Université, CNRS, INT 13005, Marseille, France; Centre IRM, Marseille 13005, France; Aix-Marseille Université, CNRS, INT 13005, Marseille, France; Aix-Marseille Université, CNRS, LPL, Aix-en-Provence 13100, France; Aix-Marseille Université, CNRS, INT 13005, Marseille, France; Aix-Marseille Université, CNRS, LPC 13331, Marseille, France

**Keywords:** cerebellum, error monitoring, fMRI, forward modeling, speech production

## Abstract

An event-related functional magnetic resonance imaging study examined how speakers inspect their own speech for errors. Concretely, we sought to assess 1) the role of the temporal cortex in monitoring speech errors, linked with comprehension-based monitoring; 2) the involvement of the cerebellum in internal and external monitoring, linked with forward modeling; and 3) the role of the medial frontal cortex for internal monitoring, linked with conflict-based monitoring. In a word production task priming speech errors, we observed enhanced involvement of the right posterior cerebellum for trials that were correct, but on which participants were more likely to make a word as compared with a nonword error (contrast of internal monitoring). Furthermore, comparing errors to correct utterances (contrast of external monitoring), we observed increased activation of the same cerebellar region, of the superior medial cerebellum, and of regions in temporal and medial frontal cortex. The presence of the cerebellum for both internal and external monitoring indicates the use of forward modeling across the planning and articulation of speech. Dissociations across internal and external monitoring in temporal and medial frontal cortex indicate that monitoring of overt errors is more reliant on vocal feedback control.

## Introduction

Several phenomena indicate that speakers inspect their utterances for errors. The most obvious evidence for this is that speakers can interrupt and correct themselves (self-repairs, Levelt 1983) or accurately report having committed an error (Postma and Noordanus 1996). Errors are sometimes interrupted or repaired almost immediately after they start to be pronounced, at a velocity indicating that error detection and repair had already been prepared internally, before the error was even audible (Levelt 1983; Hartsuiker and Kolk 2001). Moreover, certain types of errors, such as taboo or nonwords, occur below chance when they would be considered as inappropriate utterances (Baars et al. 1975; Nooteboom and Quené 2008). This indicates that the monitor can filter out impending errors before articulation, thus lending further support to the notion that monitoring may also take place internally. Despite the consensus regarding the existence of both inner and external error monitoring processes, their cognitive and neural basis remains contentious (see Lind and Hartsuiker 2020; Gauvin and Hartsuiker 2020; and Nozari 2020 for reviews). Here we aimed at better characterizing the presence of 3 different monitoring mechanisms invoked to account for both inner and external monitoring, namely 1) “comprehension-based monitoring” with neural correlates in temporal cortex, 2) “forward modeling” with neural correlates in the cerebellum, and 3) “conflict-based monitoring” with neural correlates in medial frontal cortex. To this end, we used event-related functional magnetic resonance imaging (fMRI) during an overt production task eliciting speech errors.

## Temporal Cortex and Comprehension-Based Monitoring

An influential view has been that speakers rely on speech comprehension processes to detect errors (Levelt 1983; Levelt et al. 1999; Hartsuiker and Kolk 2001; Roelofs 2020). A speaker’s own phonologically encoded internal representations and audible speech utterances would be the input of an inner and external channel, respectively, feeding into the very processing loops used when perceiving speech produced by others. This cognitive account fitted nicely with the neurobiological proposal linking monitoring processes to activity in regions of the auditory cortex (Indefrey and Levelt 2004), which was based on the observation of enhanced bilateral activation of posterior superior temporal gyrus (pSTG) in conditions requiring increased speech monitoring (e.g., manipulated auditory feedback, Hirano et al. 1997; auditory hallucinations, Shergill et al. 2000). Other models implement the reliance on speech perception for error detection as a feedback circuit comparing auditory perception with an internal auditory target, and the proposed locus of this comparison is also pSTG (e.g., Golfinopoulos et al. 2010) or the neighboring region sylvian fissure at the parietotemporal boundary (SPT) (e.g., Hickok 2012). However, a recent review and meta-analysis of 17 studies argued to support the implication of the pSTG in monitoring concluded that existing neuroimaging evidence is insufficient to make such an argument (e.g., Meekings and Scott 2021). In particular, there was a mismatch between the pSTG regions proposed as responsible for error detection in the previous literature and the regions identified in an activation likelihood estimate analysis. Also, the studies themselves were found to be methodologically and theoretically inconsistent with one another. In addition, none of the studies on which the models were built was actually based on natural speech errors, but rather on feedback alterations. Hence, it remains an open question whether the pSTG has a role in the monitoring of true speech errors.

## Cerebellum and Forward Modeling

The involvement of the cerebellum has been reported in studies involving manipulations of participants’ auditory feedback to their own speech (e.g., distorted or noisy feedback, Christoffels et al. 2007; Tourville et al. 2008), verbal fluency (e.g., produce as many words as possible beginning with “s,” Leggio et al. 2000), and error priming during speech production (e.g., “tax coal” priming the target “cap toast” into the error “tap coast,” Runnqvist et al. 2016). To understand this cerebellar involvement for speech production, one can turn to what is known about the monitoring of nonverbal actions. The cerebellum has been ascribed a crucial role in the monitoring of motor actions through the theoretical construct of forward modeling (also labeled “internal modeling” or “predictive coding”). In a forward modeling framework, the correction of motor commands is ensured by producing expectations of the commands’ sensory consequences before their output is effective as physical actions (i.e., through corollary discharges or efference copies; McCloskey 1981; Jeannerod 1988; Wolpert et al. 1995). Cerebellar activity, particularly in the posterior lobules, is modulated by the predictability of the consequences of self-generated movements (Imamizu et al. 2000; Blakemore et al. 2001). Hence, the cerebellum has been proposed as an important center of this forward modeling of motor actions (Imamizu et al. 2000; Blakemore et al. 2001; Miall and King 2008).

The hypothesis of cerebellar forward modeling has also been incorporated into theories and empirical investigations of mental activities, including language processing (Ito 2008; Strick et al. 2009; Desmond and Fiez 1998; Pickering and Garrod 2013; Hickok 2012; Lesage et al. 2017; Argyropoulos 2016). For example, Ito (2008) proposed to extend the domain of forward models from sensorimotor actions to mental activities based on a review of anatomical (i.e., appropriate neural wiring between the cerebellum and the cerebral cortex), functional (appropriate mental activity in the cerebellum), and neuropsychological data (the association of some mental disorders with cerebellar dysfunction). In line with this proposal, it has been shown that a gradient within the posterolateral cerebellum supports cognitive control of both concrete, proximal actions (motor-adjacent subregions) and abstract future processing (motor-distal subregions, e.g., D’Mello et al. 2020). Several theoretical models of the motor control of speech incorporate some form of forward modeling (i.e., Guenther et al. 2006; Tourville and Guenther 2011; Hickok 2012, 2014; Tian and Poeppel 2010). For example, Golfinopoulos et al. (2010) propose that auditory feedback control would be complemented by a cerebellar module (superior lateral cerebellum) and a feedforward control subsystem mediated by a transcerebellar pathway (anterior paravermal parts of the cerebellum). Hickok (2012) proposes that the cerebellum is in charge of the comparison (coordinate transform) between auditory and motor targets at the phonetic encoding stages of speech production. The integration of the cerebellum in these models is based on evidence from feedback manipulations as discussed previously (e.g., Ghosh et al. 2008) and on the role of the cerebellum in ataxic dysarthria studies (e.g., Ackermann et al. 1992). A less explored hypothesis states that linguistic levels of processing that are beyond speech motor control are also monitored through forward models (Pickering and Garrod 2013). Furthermore, this psycholinguistic proposal has not been neurobiologically specified. However, given the increasing evidence of a role of the cerebellum in cognitive processing, an extension of the mechanisms operating on speech motor aspects to language processing proper is conceivable. One study has reported an increase in the production of phonological substitution errors after repetitive transcranial magnetic stimulation to the right posterolateral cerebellar Crus I (e.g., Runnqvist et al. 2016). Hence, this study suggests a direct involvement of the posterior cerebellum in speech monitoring beyond articulatory aspects. However, among others, open questions that remain are whether this type of monitoring is applied during planning or articulation and whether the same or different parts of the cerebellum would be involved for monitoring inner versus overt speech.

## Medial Frontal Cortex and Conflict-Based Monitoring

The involvement of several areas in the medial frontal cortex such as the presupplementary motor area (pre-SMA) and the anterior cingulate cortex (ACC) has been reported in studies investigating error related processing in language production (Gauvin et al. 2016; De Zubicaray et al. 2001; Möller et al. 2007). These areas are the same ones that have been linked to error detection and conflict monitoring in domains other than language, such as in cognitive control (Botvinick et al. 2001; Nachev et al. 2005). The conflict monitoring theory holds that medial frontal structures constantly evaluate current levels of conflict and that, when a conflict threshold is passed, they relay this information on to other regions in frontal cortex responsible for control, triggering them to adjust the strength of their influence on processing. A need for greater control is thus indicated by the occurrence of conflict itself. Such theory can account both for inner and external monitoring through a single mechanism operating on a continuum of conflict on which overt errors would be the most extreme case.

The idea of conflict monitoring as a means of preventing and detecting errors has been incorporated into a model of language production (Nozari et al. 2011) that successfully simulated error detection performance in aphasic patients. Moreover, a few studies have obtained evidence for an involvement of the ACC and pre-SMA also on correctly named trials in tasks involving the presence of explicit conflict in the stimulus to be processed for language production (e.g., semantic interference inflicted by the categorical relationship between a picture to be named and a (near-) simultaneously presented distractor; De Zubicaray et al. 2001; Abel et al. 2012). However, the available evidence only bears on the involvement of medial frontal cortex in the processing of overt errors or of conflict of the type requiring the exclusion of a competing response that is directly present in the stimulus. Hence, in the context of a task without explicit conflict in the stimulus, it remains an open question whether the medial frontal cortex has a role for monitoring in the absence of overt errors.

## The Current Study

In short, 3 hypotheses about cognitive mechanisms with distinct neural correlates can be distilled from the literature related to internal and external speech error monitoring, namely comprehension-based monitoring through posterior temporal cortex, forward modeling through the cerebellum, and conflict-based monitoring through medial frontal cortex. As evidenced by our review of the literature, many questions regarding the circumstances in which these mechanisms may be at play remain open. Here we sought to fill some of these gaps by providing independent empirical support for 1) a role of the temporal cortex in the monitoring of true speech errors; 2) an involvement of the cerebellum in inner and/or external monitoring, possibly recruiting different parts of the cerebellum for different functions (posterior for speech planning and superior medial for articulation); and 3) a role of the medial frontal cortex for inner monitoring (in the absence of overt errors). We addressed these hypotheses through an event-related fMRI study designed to examine both internal and external speech error monitoring, with a zoom on temporal, cerebellar, and medial frontal regions linked to the different monitoring mechanisms discussed above.

Eleven regions of interest (ROI) were selected within these 3 broad anatomical regions (Table 1), corresponding to Montreal Neurological Institute (MNI) coordinates reported in theoretically relevant meta-analyses, models, or studies eliciting natural speech errors. In particular, our ROIs in temporal cortex correspond to pSTG regions proposed to underlie the auditory target in the DIVA model (e.g., Golfinopoulos et al. 2010) and to the region SPT that corresponds to the coordinate transform between auditory and motor targets in the HSFC model (e.g., Okada and Hickok 2006). For the cerebellum we selected 2 right posterior coordinates linked to (cognitive aspects of) language processing in the meta-analysis of Stoodley and Schmahmann (2009) as well as the coordinates corresponding to the superior medial cerebellum linked to the articulatory aspects of speech in the DIVA model (e.g., Golfinopouolous et al. 2010). Finally, for medial frontal cortex we selected the coordinates reported for ACC and pre-SMA in Gauvin et al. (2016), being the only previous study that directly contrasted overt natural speech errors and correct trials. For estimates on the right ACC and pre-SMA we used the coordinates reported by the meta-analysis of Hester et al. (2004) stemming from nonlinguistic error-related processing.

**Table 1:** MNI coordinates and references of the ROI classified by anatomical regions and monitoring account

			Comprehension-based	Forward modeling	Conflict-based
			Inner monitoring (inner speech) and external monitoring (audible speech)	Inner monitoring (linguistic dimensions) and/or external monitoring (motor dimensions)	Inner monitoring (impending errors) and/or external monitoring (overt errors)
Medial frontal cortex	ACC	roi1_ACC_L roi2_ACC_R			(−6, 20, 34) Gauvin et al. 2016 (1, −14, 39) Hester et al. 2004^*^
	Pre-SMA	roi3_Pre-SMA_L roi4_Pre-SMA_R			(−6, 8, 49) Gauvin et al. 2016 (11, −9, 53) Hester et al. 2004^*^
Cerebellum	Posterior	roi5_RCB1_R roi6_RCB2_R		(37.9, −63.7, −29.7) (12.5, −86.1, −32.9) Stoodley and Schmahmann 2009^*^	
	Superior medial	roi7_SMC_L roi8_SMC_R		(−18, −59, −22) (16, −59, −23) Golfinopoulos et al. 2010^*^	
Temporal cortex	SPT	roi9_SPT_L		(−54, −30, 14) Okada and Hickok 2006	
	pSTG	roi10_pSTG_L roi11_pSTG_R	(−64.6, −33.2, 13.5) (69.5, −30.7, 5.2) Golfinopoulos et al. 2010^*^		

Asterisks indicate meta-analysis or model-based coordinates.

**Figure 1:**
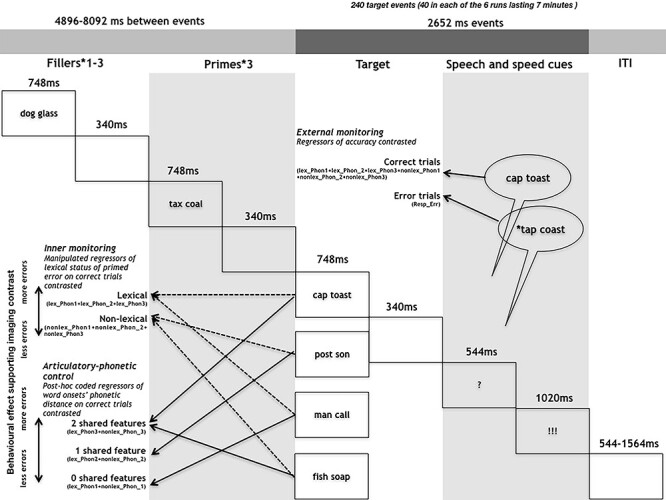
Depiction of the experimental design and procedure.

Twenty-four healthy volunteers, native speakers of French, performed an error eliciting production task while undergoing blood-oxygen-level-dependent (BOLD) imaging. Based on evidence that a majority of overt errors involve error detection and hence monitoring (Gauvin et al. 2016), external monitoring was indexed by contrasting correct trials and trials with errors. Extending previous work, internal monitoring was indexed on correct trials by manipulating the likelihood of committing an error and hence the load on speech monitoring mechanisms in 2 conditions. This was achieved by priming spoonerisms that for half of the trials would result in lexical errors (e.g., “tap coast” for the target “cap toast”) and the other half in nonlexical errors (e.g., “^*^sost ^*^pon” for the target “post son,” Fig. 1). Speakers are more error-prone when lexical rather than nonlexical errors are primed (Nooteboom and Quené 2008; Oppenheim and Dell 2008). This effect seems to be caused by a combination of context biases (inappropriate production candidates are more easily discarded, e.g., Hartsuiker et al. 2005) and of the interactive activation dynamics inherent to speech preparation (the lexical competitor would count on both a phonological and lexical source of activation compared with the nonlexical one, e.g., Dell 1986). Regardless of the cause of the effect, the rationale here is that to-be-articulated words with higher error probability should reveal an enhanced involvement of the inner monitor (Severens et al. 2012). Hence, lexical versus nonlexical error priming was contrasted to index internal monitoring.

## Methods

### Participants

The study received appropriate ethical approval (filed under id “EudraCT: 2015-A00845-344” at the regional ethical committee “Comité de Protection des Personnes Sud Méditerranée I”). Twenty-eight (18 females, 10 males) right-handed native speakers of French participated in exchange for monetary compensation. Four participants (4 males) were excluded from the analyses: 3 because of excessive head movements during the acquisition and 1 because of a misunderstanding of the task. The average age of the remaining 24 participants was 23.8 (SD 3.2). No participant reported any history of language or neurological disorders.

### Materials

Target stimuli were 320 printed French nouns (those used in Runnqvist et al. 2016) to be presented in pairs. For illustrative purposes, the examples in the text are given in English. To control for differences due to physical variance of stimuli, the same words were used across participants and conditions (albeit combined differently to prime lexical and nonlexical errors, e.g., “mole sail,” “mole fence”). Exchanging the first letters of these combinations would result in a new word pair in one case (“sole mail,” lexical error outcome) and in a nonword pair in the other case (“fole mence,” nonlexical error outcome). All combinations for which the exchange of initial phonemes resulted in new word pairs (mole sail) were used also in reversed order (sole mail). An orthographic criterion was used for selecting stimuli. To control for the variable of phonetic distance of the word pair onsets across the conditions of interest, these were coded for the degree of shared phonetic features (place and manner of articulation plus voicing), being assigned a number ranging from 0 (phonetically distant words) to 2 (phonetically close words). This was deemed necessary because with decreasing phonetic distance between onsets speakers are more likely to exchange onsets (e.g., Nooteboom and Quené 2008). We also included this variable in all analyses and we report the corresponding results in the supplementary information (Supplementary Tables 2–4 and Supplementary Fig. 1). A total of 102 pairs shared 0 features, 161 pairs shared 1 feature, and 57 pairs shared 2 features. The stimuli across the lexical and nonlexical conditions did not differ in the average amount of shared features (lexical 0.9 shared features vs. nonlexical 0.8 shared features, *P* = .47). The words in the target pairs were selected with the criterion that they should be semantically unrelated. A given participant was only presented with one combination for each word (lexical or nonlexical outcome) and was only presented with one of the words differing in only the first sound (mole or sole). During the experiment, 3 priming word pairs preceded each target word pair. The first 2 shared the initial consonants, and the third pair had further phonological overlap with the error being primed (“sun mall”—“sand mouth”—“soap mate”—“mole sail”). To induce errors, the order of the 2 initial consonants (/s/ and /m/) is different for the primes and the target. Participants were also presented with 140 filler pairs that had no specific relationship to their corresponding target pairs. One to 3 filler pairs were presented before each prime and target sequence. Thus, each participant was presented with 460 unique word combinations (80 targets of which 40 lexical and 40 nonlexical error outcome, 240 primes and 140 fillers). Each participant completed 6 experimental runs in which word pairs were repeated 3 times in different orders. Eight lists with a different randomization of the stimuli sequences were created.

### Procedure

Word pairs remained on the screen for 748 ms. Words presented for silent reading were followed by a blank screen for 340 ms. All targets and 40% of the filler items were followed by a question mark for 544 ms, replaced by an exclamation mark presented 544 ms after the presentation of the question mark and remaining for 1020 ms. Before the next trial started there was a blank screen for 544 ms in the case of filler production trials and jittered between 544 and 1564 in the case of target production trials. The jittered inter stimulus interval was generated according to an exponential function and randomized across runs (e.g., Henson 2007). Participants were instructed to silently read the word pairs as they appeared, naming aloud the last word pair they had seen whenever a question mark was presented and before the appearance of an exclamation mark. Stimulus presentation and recording of productions to be processed offline were controlled by a custom-made presentation software compiled using the LabVIEW development environment (National Instruments).

### MRI Data Acquisition

Data were collected on a 3-Tesla Siemens Prisma Scanner (Siemens, Erlangen, Germany) at the Marseille MRI Center (Centre IRM-INT@CERIMED, UMR7289 CNRS & AMU) using a 64-channel head coil. Functional images (EPI sequence, 54 slices per volume, multi-band accelerator factor 3, repetition time = 1.224 s, spatial resolution = 2.5 × 2.5 × 2.5 mm, echo time = 30 ms, flip angle = 65°) covering the whole brain were acquired during the task performance. Whole-brain anatomical MRI data were acquired using high-resolution structural *T*_1_-weighted image (MPRAGE sequence, repetition time = 2.4 s, spatial resolution = 0.8 × 0.8 × 0.8 mm, echo time = 2.28 ms, flip angle = 8°) in the sagittal plane. Prior to functional imaging, fieldmap image (dual echo gradient-echo acquisition, repetition time = 7.06 s, spatial resolution = 2.5 mm^3^, echo time = 59 ms, flip angle = 90°) was also acquired.

**Figure 2:**
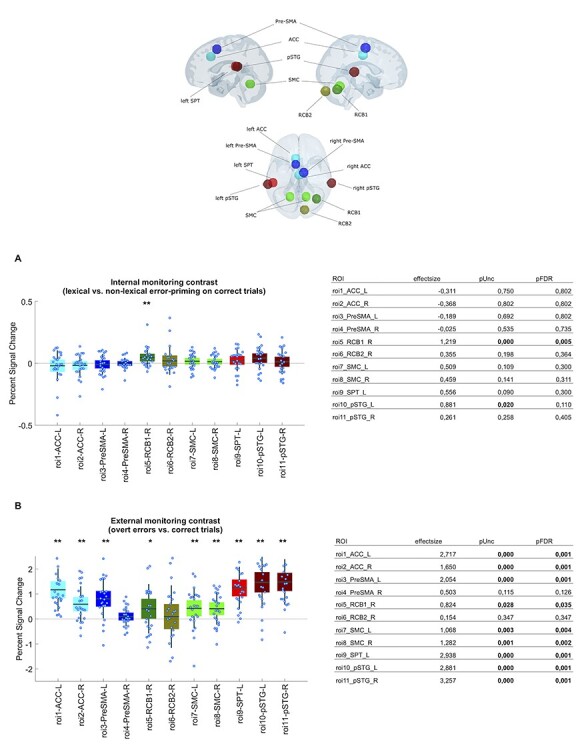
Percent signal change in the 11 predefined ROI (location in the brain in top central panel) for (*A*) the internal monitoring contrast and (*B*) the external monitoring contrast. ROIs in medial frontal cortex are represented with blue tones, ROIs in the cerebellum in green tones and ROIs in temporal cortex in red tones. The asterisks indicate significant effects <0.05 (^*^) or <0.005 (^*^^*^) after correcting for multiple comparisons using FDR.

### Behavioral Data Processing and Analyses

A person naïve to the purpose of the experiment transcribed all spoken productions and inspected and codified vocal response onsets of all individual recordings using Check-vocal (Protopapas 2007). Check-vocal is a software that allows for semiautomatic codification of the response accuracy and timing based on 2 sources of information: the speech waveform and the spectrogram. The transcriptions were scored as correct, dysfluencies, partial responses (e.g., only 1 word produced), full omissions, and erroneous productions. Errors were classified as “priming-related errors” or “other errors.” Priming-related errors included full exchanges (*mill pad* => *pill mad*), anticipations (*mill pad* => *pill pad*)*,* perseverations (*mill pad* = > *mill mad*), repaired and interrupted exchanges (*mill pad* => *pi…mill pad*), full and partial competing errors (*mill pad* => *pant milk/pant pad*), and other related errors (*mill pad* => *mad pill*). Other errors included diverse phonological substitutions that were unrelated to the priming manipulation (e.g., *mill pad* => *chill pant*/*gri..mill pad/…pant*). To assess the presence of a lexical bias and validate our assumption of a difference in monitoring load across our experimental conditions, errors were analyzed using the lme4 package (Bates et al. 2015) in R version 3.2.2 (R Development Core Team 2015). We used generalized linear mixed models (GLMM) with a binomial link function (Jaeger 2008), estimating the conditional probability of a response given the random effects and covariate values. For completeness, response times were also analyzed though we did not have any specific predictions for these. This was done using linear mixed models (LMM), estimating the influence of fixed and random covariates on the response. The summary output of the GLMM function of lme4 in R provides *P* values based on asymptotic Wald tests, which is common practice for generalized linear models (e.g., Bolker et al. 2009). In contrast, the summary output of the LMM function only provides *t*-values. Consequently, we report *P* values for error rates and *t*-values for response times. Following common practice (e.g., Fisher 1925), we take *t*-values to approximate *z*-scores and assume that absolute values above 1.96 reflect significant effects.

To assess the effect of the manipulated variable lexical status of primed errors and the control variable phonetic distance of the word pair onsets on priming-related errors, separate models were fitted for the 2 variables. The models included crossed random effects for subjects and items and the fixed factor lexicality or phonetic distance. Additional models including the same fixed and random variables were conducted on all errors and are reported in the Supplementary Tables 1–3. A histogram visualization of the response time data indicated a non-normal distribution. Therefore, log-transformed response times were modeled with mixed linear models. All models included the crossed random factors subject and item. For correct trials a first model included the fixed factor lexicality. Another model included the fixed factor shared phonetic features. A final model on all responses (i.e., both correct and incorrect trials) included the fixed factor accuracy.

### Image Processing and Analyses

The fMRI data were preprocessed and analyzed using the Statistical Parametric Mapping software (SPM12, http://www.fil.ion.ucl.ac.uk/spm/software/spm12/) on MATLAB R2018b (Mathworks Inc., Natick, MA). The anatomical scan was spatially normalized to the avg152 T_1_-weighted brain template defined by the Montreal Neurological Institute using the default parameters (nonlinear transformation). The Fieldmap images were used during the realign and unwarp procedure for distortion and motion correction. Functional volumes were spatially realigned and normalized (using the combination of deformation field, coregistered structural and sliced functional images) and smoothed with an isotropic Gaussian kernel (full-width at half-maximum = 5 mm). The Artefact Detection Tools (ART) implemented in the CONN toolbox (www.nitrc.org/projects/conn, RRID:SCR_009550) was used to define the regressors of no interest related to head movements and functional data outliers (see next section). Automatic ART-based identification of outlier scans used a 97th percentiles superior to normative samples in the definition of the outlier thresholds (global-signal z-threshold of 5 and subject-motion threshold of 0.9 mm).

For the univariate analysis on the whole brain**,** a general linear model (GLM) was generated for each subject. The GLM included, for each of the 6 runs, 7 regressors modeling response accuracy, lexical status of error priming and phonetic distance of target pair onsets: Resp_ER, lex_Phon1_CR, lex_Phon2_CR, lex_Phon3_CR, nonlex_Phon1_CR, nonlex_Phon2_CR, nonlex_Phon3_CR (CR for correct responses and ER for errors). For the contrast targeting internal monitoring, we contrasted lex_Phon1_CR, lex_Phon2_CR, and lex_Phon3_CR, with nonlex_Phon1_CR, nonlex_Phon2_CR, and nonlex_Phon3_CR. For the contrast targeting external monitoring, we contrasted Resp_ER with lex_Phon1_CR, lex_Phon2_CR, lex_Phon3_CR, nonlex_Phon1_CR, nonlex_Phon2_CR, and nonlex_Phon3_CR. For the articulatory-phonetic control, we contrasted lex_Phon1_CR and nonlex_Phon1_CR with lex_Phon3_CR, and nonlex_Phon3_CR. In the GLM, the regressors of no interest were also included using an ART text file per subject (each file described outlier scans from global signal and head movements from ART). Regressors of interest were convolved with the canonical hemodynamic response function, and the default SPM autoregressive model AR(1) was applied. Functional data were filtered with a 128 s high-pass filter. Statistical parametric maps for each experimental factor and each participant were calculated at the first level and then entered in a second-level 1-sample t-test analysis of variance (random effects analysis or RFX). All statistical comparisons were performed with a voxelwise threshold of *P* < .001 and a cluster extent threshold of 25 voxels. For the univariate analysis on ROIs, 11 anatomical ROIs were created based on the previous literature (Table 1). ROIs with a MNI coordinates center and a 10-mm-radius were created using the MarsBar SPM toolbox (Brett et al. 2002) and applying a mask that only extracted voxels pertaining to gray matter. For a given ROI mask and on the basis on unsmoothed functional images, we extracted each subject’s percent signal changes using MarsBar software (http://marsbar.sourceforge.net/). Percent signal changes were computed from canonical events using a MarsBar’s function called “event_signal” (with “max abs” option) and averaged across voxels within a ROI. From each contrast (“internal monitoring”, “external monitoring,” and “articulatory-phonetic control”), we obtained a vector of 24% signal changes (1 per subject) per ROI (n = 11). For each ROI, we performed permutation tests (from Laurens R Krol, see https://github.com/lrkrol/permutationTest) to compare the distribution of the percent signal changes to the null hypothesis (normal distribution). Statistical tests were conducted using 2000 permutations and false discovery rate (FDR) was used to correct for multiple comparisons (Benjamini and Hochberg 1995).

## Results

Out of the 5760 target trials across all participants, 706 resulted in errors (12.3%, mean standard error (MSE) 0.4, SD 32.8), of which 155 (2.7%, MSE 0.2, SD 16.2) were related to the priming manipulation. For the subset of 155 priming-related errors, more errors were made in the lexical outcome condition (3.9%, MSE 0.4, SD 19.4) than in the nonlexical outcome condition (1.5%, MSE 0.2, SD 11.9; *P* < .001; Table 2 A). This validates the assumption that, also in the present dataset, the lexical condition was more error prone and required more monitoring. As in the previous literature, no significant differences were observed in the response times between the lexical (419 ms) and nonlexical (417 ms) outcome conditions (e.g., Hartsuiker et al. 2005; Runnqvist et al. 2016). Replicating previous findings (e.g., Gauvin et al. 2016), correct trials (418 ms) were produced faster than trials with errors (506 ms; Table 2 B).

**Table 2:** Summary of the GLMM of priming-related errors (A) and the LMMs on response times (RTs) (B)

**A: Errors**	**Effect estimate**	**Std.err.**	***z*-value**	***P* value**
Intercept	−3.70	0.20	−18.40	<.001
Lexical status (nonlexical)	−1.06	0.22	−4.85	<.001
**B: RTs**	**Effect estimate**	**In ms**	**Std.err.**	***t*-value**
Intercept	5.98	419	0.04	147 0.77
Lexical status (nonlexical)	−0.003	−2	−0.01	0.269
Intercept	6.17	504	0.04	149.99
Accuracy (error)	−0.19	−86	0.01	−13.26

Using MNI coordinates reported in the previous literature (Table 1), we examined percent signal change for our 2 contrasts in 11 predefined ROIs located in temporal, cerebellar, and medial frontal regions. A ROI in the right posterior cerebellum was involved both in the contrast targeting external monitoring (q = 0.035, d = 0.82) and in the internal monitoring of words (q = 0.005, d = 1.22; Fig. 2). Furthermore, external monitoring was also linked to bilateral superior medial cerebellum (left q = 0.004, d = 1.07; right q = 0.002, d = 1.28), bilateral ACC (left q < 0.001, d = 2.72; right q < 0.001, d = 1.65), left pre-SMA (q < 0.001, d = 2.05), region SPT (q < 0.001, 2.94), and bilateral pSTG (left q < 0.001, d = 2.88; right q < 0.001, d = 3.26).

To follow up on the potential differences in internal and external monitoring, we directly compared the external monitoring contrast with the internal monitoring contrast. The effects were larger for the former compared to the latter in bilateral superior medial cerebellum (left q = 0.022, d = −0.94; right q = 0.003, d = −1.14), bilateral ACC (left q < 0.001, d = −2.41; right q < 0.001, d = −1.51), left pre-SMA (q < 0.001, d = −1.88), region SPT (q < 0.001, d = −2.61), and bilateral pSTG (left q < 0.001, d = −2.56; right q < 0.001, d = −2.90).

**Figure 3:**
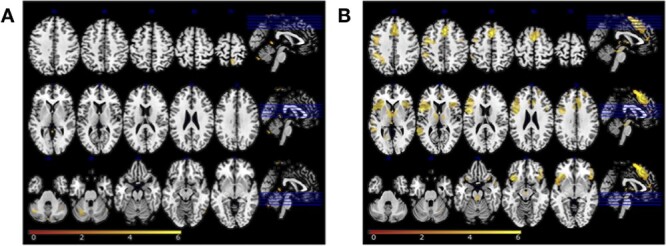
RFX results on the BOLD response of internal monitoring (lexical vs. nonlexical error priming; panel *A*) and external monitoring (errors vs. correct trials; panel *B*). Statistical *t*-maps are overlaid on MNI cortex slices (5 axial slices and 1 sagittal slice per line) using a voxelwise threshold of *P* < .001 and an extent threshold of 25 voxels.

To examine the specificity of the findings from the ROI analyses, we also conducted a whole-brain analysis (Table 4 and Fig. 3). In the internal word monitoring contrast, only the BOLD response of a cluster in the left posterior cerebellum (lobule VI) survived the correction for multiple comparisons. For the contrast targeting external monitoring, significant clusters of differential BOLD response were observed in frontal, medial frontal, temporal, insular, and parietal regions in cortex as well as regions in basal ganglia. Table 3 summarizes all the analyses that were carried out.

**Table 3:** Summary of the different analyses conducted

	Analysis	Contrasted variables	Purpose
Behavioral data	Generalized mixed linear model on priming-related errors	Lexical versus nonlexical error priming	Validate monitoring load assumption underlying imaging contrast
	LMM on response times	Lexical versus nonlexical error priming	
		Errors versus correct trials	
Brain data	Analysis on percent signal change in 10 mm spherical predefined ROI	Lexical versus nonlexical error priming	Index internal monitoring
		Errors versus correct trials	Index external monitoring
		(lexical vs. nonlexical error priming) versus (errors vs. correct trials)	Compare internal and external monitoring
	Univariate whole brain analysis on BOLD response	Lexical versus nonlexical error priming	Assess specificity of ROI findings
		Errors versus correct trials	

**Table 4:** Results of the whole-brain analyses of the BOLD response of the external (A) and internal (B) monitoring contrasts

A. External monitoring (errors vs. correct trials)			MNI coordinates		
Region label	Extent	*t*-value	*x*	*y*	*z*
**L superior medial gyrus**	1244	8.064	−3	22	48
**L posterior–medial frontal**	1244	5.908	−3	2	63
**R ACC**	1244	6.373	8	27	28
L ACC	36	3.970	0	44	13
**L inferior frontal gyrus (pars opercularis)**	2343	7.592	−48	4	16
**L inferior frontal gyrus (pars orbitalis)**	2343	7.265	−35	29	−2
**R inferior frontal gyrus (pars orbitalis)**	542	5.685	45	39	−10
R inferior frontal gyrus (pars triangularis)	51	5.742	48	14	28
**R superior frontal gyrus**	135	5.538	20	52	36
**L precentral gyrus**	2343	6.923	−45	−4	51
					
**L middle temporal gyrus**	195	7.248	−58	−46	8
L middle temporal gyrus	65	4.829	−60	−21	1
					
**L thalamus proper**	349	6.590	−10	−6	6
**R pallidum**	349	5.354	13	4	3
Brain stem	71	6.120	0	−29	−17
L dorsal caudal	28	4.828	−5	−14	−12
					
**R insula lobe**	542	5.756	35	24	3
					
**L inferior parietal lobule**	334	5.662	−45	−41	41
					
					
R cerebellum (VI)	26	4.109	30	−61	−30
					
**B. Internal monitoring (lexical vs. nonlexical error priming)**					
					
**L cerebellum (VI)**	160	4.994	−28	−66	−27
R cerebellum (VIII)	36	3.917	35	−51	−42
					
R precuneus	51	4.644	5	−56	71
					

Local maxima of BOLD response separated by >20 mm. Regions were automatically labeled using the Anatomy Toolbox atlas. *x*, *y,* and *z* = MNI coordinates in the left–right, anterior–posterior and inferior–superior dimensions, respectively. All peaks are significant at a voxelwise threshold of *P* < .001 (extent threshold = 25 voxels). Peaks that are significant at a cluster threshold of *t* < .05 with an FDR correction for multiple comparisons are marked with bold fonts. L = left, R = right.

In summary, both the contrast targeting internal monitoring of words and the contrast targeting external monitoring of errors revealed a differential percent signal change in the right posterior cerebellum. The latter contrast also revealed a differential percent signal change in superior medial cerebellum and of temporal and medial frontal regions.

## Discussion

In this study, we explored the neural basis of the cognitive mechanisms that allow speakers to monitor their speech, both internally during planning and externally during articulation. Concretely, we aimed at answering 1) whether the pSTG has a role in the monitoring of actual speech errors indicating comprehension-based monitoring; 2) whether the cerebellum would be involved in inner monitoring (posterior) and/or external monitoring (superior medial), indicating forward modeling; and 3) whether the medial frontal cortex would be involved in the presence of inner monitoring load, indicating conflict-based monitoring. In the following we discuss how the results answered these questions.

### Temporal Cortex and Monitoring of Speech Errors

All 3 ROIs in temporal cortex (bilateral pSTG and SPT) showed a differential percent signal change for speech errors compared to correct trials. Hence, some form of comprehension-based monitoring likely takes place in the case of overt speech errors (or more strongly for speech errors compared with correct utterances). The current study cannot answer whether such comprehension-based monitoring is carried out through speech comprehension processes directly, through feedback control processes (comparing auditory percepts and targets) or in the form of increased response for unexpected input (and thus connected to the cerebellar forward modeling that will be discussed later on). Importantly, this is the first study showing a role of pSTG/SPT for an overt speech production task involving the articulation of natural speech errors. The whole-brain analysis of the BOLD response for the contrast targeting external monitoring revealed 2 clusters peaking in the left middle temporal gyrus and in the left inferior parietal lobule, respectively, given their extent, that likely comprise the voxels targeted by the ROI coordinates. Thus, the whole-brain analysis seems to further confirm the ROI results and also sheds light on the fact that these results are not very specific as rather large portions of temporal and parietal cortex are differentially active for errors compared to correct trials.

### Cerebellum Involved in both Internal and External Monitoring

The contrast targeting internal monitoring showed a differential percent signal change in a region in the right posterior cerebellum that has been attributed an important role in the forward modeling of self-generated actions (e.g., Imamizu et al. 2000; Blakemore et al. 2001; Ito 2008; Miall and King 2008; Strick et al. 2009). To our knowledge, this is the first time that the involvement of the cerebellum in the internal monitoring of an unambiguously linguistic aspect of language production has been reported. While previous studies have reported an involvement of the cerebellum for articulatory–acoustic aspects of speech, here the involvement was modulated by lexical information, a level of language processing that is distinct from the sensory–motor aspects of speech. One possibility is that this occurs because in language use sound and meaning always cooccur. Over time, this arguably leads the 2 dimensions to form an interconnected distributed representation (Strijkers 2016; Fairs et al. 2021). This holistic format of linguistic representations would entail that sound and meaning dimensions would become active in parallel both when producing and understanding speech, hence over time also sharing processing dynamics. In this way, motor control processes could be directly applied to any level of language processing. Another, not mutually exclusive, possibility is that all self-generated actions, whether motor or mental, may be supervised through forward modeling enabled by cerebellar connections to different areas of cortex (Ito 2008; Strick et al. 2009). The cerebellum would generate the prediction of the sensory or mental consequences of the action (efference copying), whereas the cortical region in question would be in charge of inhibiting the neural response that the action is expected to generate. In the case of language, the modeling of different levels of linguistic representation might result in reafference cancelation in different areas of cortex. Regardless the exact mechanism, the link between cerebellum activity and a processing level in principle distant from articulation calls for an extended role of the cerebellum (i.e., beyond speech motor control) in current models (Golfinopoulos et al. 2010; Hickok 2012).

Secondly, the contrast targeting external monitoring showed a differential percent signal change of the same right cerebellar region as internal monitoring and also a differential percent signal change bilaterally of the ROIs located in the superior medial cerebellum. This latter region has been linked to articulatory difficulties such as ataxic dysarthria and hence speech motor control troubles. An interesting possibility is that the posterior cerebellar activation might be especially due to the lexical and fluent errors (being more similar to the effect of the inner monitoring contrast) and the superior medial cerebellar activation might be especially due to nonlexical or more dysfluent errors. Unfortunately, however, while we are able to pinpoint an exact level of processing for our inner monitoring contrast thanks to the error priming manipulation, for the errors this was not possible because overt nonlexical errors are so rare that not all participants have observations for these. For the same reason, the errors included in the external monitoring contrast are also diverse in nature (i.e., all errors were pooled together and contrasted with correct responses). Finally, given that in the external monitoring contrast the cerebellar activation was accompanied by pSTG/SPT activation, a parsimonious assumption is that the less predictable auditory response associated with an error led to a lowered reafference cancelation.

Turning to the whole-brain analyses of the BOLD response, unexpectedly, a region in the left posterior cerebellum was differentially activated in the contrast targeting internal monitoring. With the aim of guiding future hypotheses concerning language processing and monitoring in the cerebellum we visualized the peak coordinates of the cluster in an atlas viewer of the cerebellum, SUIT (e.g., Diedrichsen 2006), allowing to overlay different task contrast maps onto an anatomical template. Nine contrasts overlapping with the observed region could be more or less directly linked to the current task contrast through the notion of (verbal) working memory (object 2 back, object 2 back+, verbal 2 back, and verbal 2 back+); prediction outcome (true, violated, and scrambled predictions); and response difficulty (easy and medium responses). Broadly, all 3 groupings are consistent with the notion of increases in monitoring load engaging processes of forward modeling (e.g., Runnqvist et al. 2016). More generally, this result shows that the left cerebellum should not be neglected in studies of language where it is often assumed that cerebellar contributions to language processing are right lateralized. Furthermore, the results of the whole-brain analysis highlight the fact that cerebellar activity is elusive and may go undetected without an appropriate task (sufficiently demanding), analysis of different task stages (early vs. late stages, e.g., Imamizu et al. 2000), or statistical approach (such as a ROI approach, see Johnson et al. 2019, for an extended argumentation).

### Medial Frontal Cortex for External Monitoring

For the contrast targeting external monitoring, we observed a differential percent signal change bilaterally for ACC as well as for left pre-SMA in our ROI analyses. Previous studies contrasting errors and correct trials have reported a similar pattern and this has been interpreted in terms of conflict-based monitoring (Gauvin et al. 2016; Riès et al. 2011). However, no such differential percent signal change in medial frontal cortex was observed for inner monitoring, and when comparing both contrasts directly the difference was significant (i.e., more ACC and pre-SMA percent signal change in external compared with internal monitoring). Consistent with this, the whole-brain analyses of the BOLD response revealed 2 very broadly extended clusters in the left superior medial gyrus and 1 in the right ACC only for the external monitoring contrast. As for temporal cortex, given their size, they are likely to comprise the voxels targeted by our ROI but again show that the activation is much more extended than these. While the current study cannot add much anatomical specificity to the debate, the dissociation of medial frontal activity for the internal and external monitoring contrasts hints that conflict is not the mechanism behind the differential percent signal change and BOLD response. One possibility is that the involvement of the medial frontal cortex observed here is related to a vocal cognitive control network shared across primates as proposed recently by Loh et al. (2020). These authors argue that, across primates, area 44 is in charge of cognitive control of orofacial and nonspeech vocal responses, and the midcingulate cortex is in charge of analyzing vocal nonspeech feedback driving response adaptation. Furthermore, the cognitive control of human-specific speech vocal information would require the additional recruitment of area 45 and pre-SMA. In this framework, it would not be the conflict that generates the ACC and pre-SMA percent signal change and BOLD response observed here but rather the feedback provided through the articulated error. An advantage of this feedback-based network account of vocal cognitive control is that it also predicts the BOLD response clusters in the left inferior frontal gyrus that we observed (while a conflict account would rather predict activation in dorsolateral prefrontal cortex, e.g., MacDonald et al. 2000).

In summary, monitoring for errors during speech production seems to rely on a broad network of brain regions that can be linked to different monitoring mechanisms (e.g., modeling of self-generated actions, cognitive control, and sensorial perception) in accordance with previous findings reported in the literature. Importantly, however, this is the first time that multiple monitoring mechanisms are investigated simultaneously in the context of both speech planning and articulation, allowing us to show that certain regions (pSTG, SPT, ACC, and pre-SMA) seem to be implicated preferentially in the context of overt errors and thus seemingly more dependent on the sensorial feedback. However, perhaps the most striking result is that the same posterior part of the right cerebellum is involved both in inner and external monitoring, a finding that is challenging for all current brain models of language production. The results reported here show the importance of adopting a broad approach when addressing complex cognitive processes like error monitoring of multidimensional representations (language) at the service of a combined mental and motor action (speaking). Previous studies may have failed to detect the involvement of certain monitoring regions because only 1 ROI or only 1 manipulation of monitoring demands were examined at the same time. To be addressed in future research is whether these different functional regions are competitively or collaboratively interconnected or whether they are instances of partially redundant cognitive mechanisms that, in an analogous way to redundant input in the environment, could serve to increase the likelihood of detecting and correcting errors in noisy neural communication channels (Barlow 2001).

## Supplementary Material

supplementary_materials_Runnqvist_et_al_CerCorComms_tgab038
